# Clinical Course and Treatment of Children and Adolescents with The Preexcitation Syndrome − Own Studies

**DOI:** 10.34763/devperiodmed.20182202.113122

**Published:** 2018-06-30

**Authors:** Aleksandra Stasiak, Katarzyna Niewiadomska-Jarosik, Piotr Kędziora

**Affiliations:** 1 Department of Pediatric Cardiology and Rheumatology, Medical University of Lodz, Lodz Poland

**Keywords:** child, preexcitation syndromes, WPW syndrome, dziecko, zespoły preekscytacji, zespół WPW

## Abstract

**Introduction:**

Essentially, preexcitation syndrome is the presence of an accessory pathway in the heart, which can lead to serious consequences, ranging from atrioventricular reentrant tachycardia to sudden cardiac death. Wolff-Parkinson-White syndrome is the most common preexcitation syndrome.

**Aim of the study:**

The aim of the study was to evaluate the clinical course of the disease, as well as the treatment of children and adolescents hospitalized in the Department of Pediatric Cardiology in the years 2008-2015.

**Materials and methods:**

The study was carried out in 45 children (62 % male, 38% female; the mean age 11 years). During the study we analyzed 12-lead ECG, 24-hour Holter ECG, echocardiography and the cycloergometric exercise test. The results of treatment were also discussed.

**Results:**

Apart from the typical features of preexcitation, the most prevalent abnormality found in ECG was atrioventricular reentrant tachycardia. In 24-hour Holter ECG the most frequently detected disorders were premature ventricular beats and premature atrial contractions. Structural heart defects were detected in 8.9% of the children. The cycloergometric exercise test was positive in 8.9% of patients. The mean duration of symptoms before the diagnosis was 2.5 years. 25% of the patients were asymptomatic. 42.2% of the children needed antiarrhythmic therapy, while 44.4% had accessory pathways ablated.

**Conclusions:**

The most common symptom of preexcitation in the study group were heart palpitations. The most frequent type of arrhythmia in children with preexcitation syndrome was orthodromic atrioventricular reentrant tachycardia. For the majority of older children ablation of the accessory pathway was a recommended form of treatment. In younger children the standard preventive pharmacological treatment was applied for 6 to 12 months.

Charakterystyczną cechą zespołów preekscytacji jest obecność dodatkowej, nieprawidłowej drogi przewodzenia w sercu. Najczęstszym typem zespołu preekscytacji jest zespół Wolffa-Parkinsona-White’a.

## Streszczenie

**Cel pracy:**

Ocena przebiegu klinicznego oraz postępowania terapeutycznego u dzieci z zespołem preekscytacji hospitalizowanych w Klinice Kardiologii Dziecięcej w latach 2008-2015.

**Materiał i metody:**

Retrospektywna ocena danych klinicznych zebranych przez okres 7 lat obejmuje grupę 45 dzieci (62% chłopców, 38% dziewczynek; średni wiek 11 lat). U dzieci z zespołem preekscytacji z badanej grupy wykonano 12-odprowadzeniowy zapis EKG, 24-godzinny zapis EKG metodą Holtera, badanie echokardiograficzne serca, a u dzieci starszych i młodzieży przeprowadzono także próbę wysiłkową na ergometrze rowerowym wg protokołu James’a. Oceniono również postępowanie terapeutyczne.

**Wyniki:**

W zapisie EKG oprócz typowych cech preekscytacji najczęstszą nieprawidłowością był częstoskurcz przedsionkowo-komorowy. W 24-godzinnym Holterze EKG najczęściej wykrywanymi zaburzeniami rytmu były przedwczesne pobudzenia komorowe oraz przedwczesne pobudzenia nadkomorowe. W badaniu echokardiograficznym wrodzone wady serca stwierdzono u 4 dzieci (8,9%). U 8,9% dzieci wynik próby wysiłkowej był dodatni. Średni czas postawienia rozpoznania wyniósł 2.5 roku. 25% pacjentów nie wykazywało żadnych objawów choroby. U 42,2% dzieci wdrożono leczenie antyarytmiczne, a 44,4% pacjentów zakwalifikowano do zabiegu ablacji.

**Wnioski:**

W badanej grupie najczęstszym objawem zespołu preekscytacji były kołatania serca. Najczęstszym typem arytmii był ortodromowy częstoskurcz przedsionkowo-komorowy. W grupie starszych dzieci zalecaną formą terapii była ablacja drogi dodatkowej. U młodszych dzieci zgodnie ze standardami zastosowano prewencyjną terapię antyarytmiczną w okresie od 6 do 12 miesięcy. Długi okres czasu potrzebny do postawienia rozpoznania wskazuje na konieczność podjęcia dalszych badań w zakresie problemów związanych z zespołem preekscytacji.

## Introduction

Preexcitation syndromes are a condition increasingly diagnosed also in children. The essence of the disease is the presence of an additional, anomalous pathway in the heart, which conducts atrial impulses to the ventricles faster than the normal conductive system (atrioventricular node), potentially providing a substrate for atrioventricular reentrant tachycardia. The most common type of preexcitation syndrome is Wolff-Parkinson-White Syndrome (WPW), which affects about 2/1000 people. While many of those patients remain asymptomatic, Wolff-Parkinson-White syndrome may also manifest itself as atrioventricular reentrant tachycardia (AVRT), as ventricular fibrillation, or may even lead to sudden cardiac death.

The aim of the study: to examine the symptoms and clinical course, as well as the treatment of children and adolescents with preexcitation syndrome hospitalized in the Department of Pediatric Cardiology.

## Materials and methods

The study group consisted of 45 children (28 boys (62%), 17 girls (38%) with preexcitation syndrome hospitalized in the Department of Pediatric Cardiology in the years 2008-2015. The mean age was 11 years (girls 11.6 years, boys 10.5 years). The youngest patient was admitted to hospital at the age of 8 days and the oldest one at the age of 17.5 years.

**Fig. 1:**
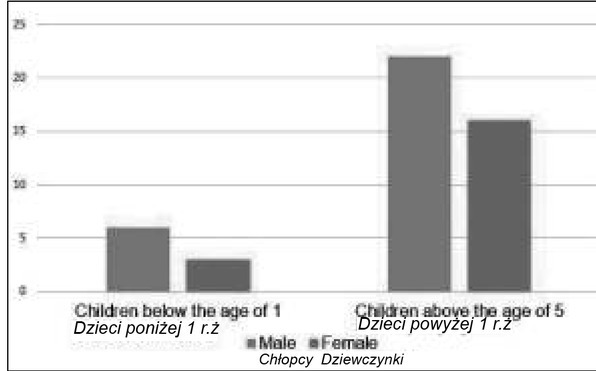
Division of the study group by age and gender. Ryc. 1. Podział badanej grupy według wieku i płci.

Due to the differences in the therapeutic process and the possibility of spontaneous loss of electrical activity of an accessory pathway within the first year of life, the study group was further divided into a group composed of patients under the age of one year (20% of the study group: 6 boys and 3 girls; mean age 7 months) and a group of children over 5 years of age, which constituted 80% of the study group, with the mean age of 13.5 years (Figure 1). In the group of patients hospitalized for the first time there were no patients between 1 and 5 years of age.

One child in the study group (2.2%) had a family history of Wolff-Parkinson-White syndrome (the father and brother of the patient have also been diagnosed with Wolff-Parkinson-White syndrome).

In one child the symptoms of preexcitation were probably present in fetal life in the form of atrioventricular tachycardia. Obstetricians decided to perform Caesarean section prematurely. The child remained under cardiological observation for the first year of life, then no specialist care was provided until the recurrence of atrioventricular reentrant tachycardia at the age of nine.

During the study each child had at least one 12-lead electrocardiogram (ECG), one 24-hour Holter ECG recording and one echocardiogram performed. 24 children from the group of patients over the age of five years (53.3%) performed the cycloergometric exercise test using the James protocol. A negative result of the exercise test included abrupt disappearance of preexcitation or no impact of physical exercise on preexcitation. A positive exercise test included the occurrence or intensification of arrhythmias, such as tachycardia with narrow or wide QRS complex, or additional premature ventricular/atrial contractions. In 5 children (11.1%) the diagnostic process was complemented by 24-hour blood pressure monitoring and an orthostatic test.

## Results

**Fig. 2:**
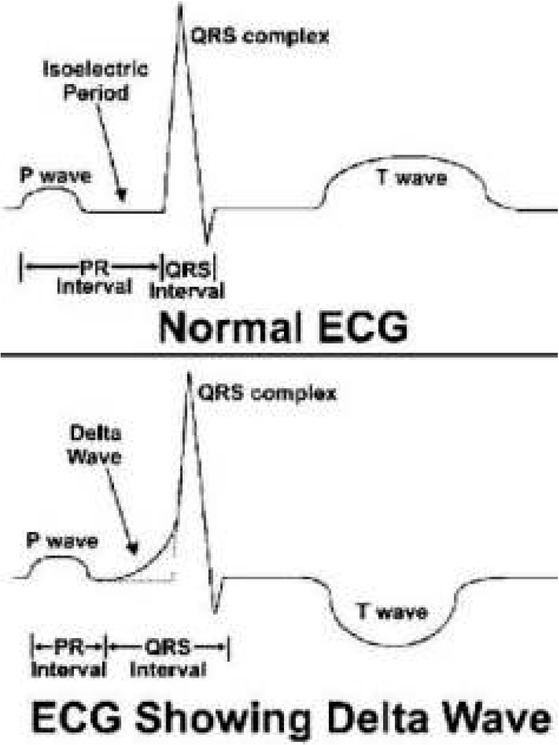
Diagram presenting normal ECG and ECG with characteristic features of preexcitation syndrome [*Ventricular pre-excitation: WPW and LGL syndromes*. [stan z 12.03.2014] http://www.frca.co.uk/article.aspx?articleid=100691#faq] Ryc. 2 Prawidłowy zapis EKG oraz zapis EKG z cechami charakterystycznymi dla zespołu preekscytacji.

Apart from the typical features of preexcitation (Figure 2), ECG revealed that 21 children (44.4%) had abnormal cardiac axis deviation. 15 children (33.3%) – i.e. 14 children in the group older than five years and 1 child in the group under the age of one year – had left axis deviation, which may be associated with the Wolff-Parkinson-White syndrome. The most common arrhythmia recorded in the ECG of 21 children (46.7%) was atrioventricular reentrant tachycardia (Table I).

**Table I:** Abnormalities registered in the 12-lead ECG. Tabela I. Zmiany rejestrowane w 12-odprowadzeniowym spoczynkowym zapisie EKG.

Division of patients according to age *Podział pacjentów ze względu na wiek*		
Abnormalities registered in the 12-lead ECG *Nieprawidłowości rejestrowane* *w 12-odprowadzeniowym spoczynkowym zapisie EKG*	Patients over the age of 5 (36 children) *Pacjenci powyżej 5 r.ż. (36 dzieci)*	Patients under the age of 1 (9 children) *Pacjenci poniżej 1 r.ż. (9 dzieci)*
Leti axis deviation *Lewogram*	38.9%	11.1%
Right axis deviation *Prawogram*	−	66.6% − a correct feature in this age group.
Atrioventricular reentrant tachycardia *Nawrotny częstoskurcz* *przedsionkowo-komorowy*	36.1%	88.9%
Apparent preexcitation *Jawna preekscytacji*	80.5%	55.6%
Intermittent preexcitation *Intermitująca preekscytacja*	16.7%	44.4%
Latent preexcitation *Utajona preekscytacja*	2.8%	−

In 24-hour Holter ECG, apart from the typical features of preexcitation, the most frequent disorders were premature ventricular contractions - recorded in 15 children (33.3%), and premature atrial contractions, which also occurred in 15 children (33.3%). Less frequently recorded abnormalities included sinus tachycardia (in 6 patients (13.3%)), episodes of a second-degree sinoatrial block in 3 children (6.7%) and episodes of a second-degree atrioventricular block type II 2:1(in 1 child (2.2%) (Table II). Atrioventricular reentrant tachycardia was recorded in 24-hour Holter ECG in 2 children (4.4%).

**Table II:** Arrhythmias registered in the 24-hour Holter ECG. Tabela II. Zaburzenia rytmu zarejestrowane w 24-godzinnym zapisie EKG metodą Holtera.

Division of patients according to age *Podział pacjentów ze względu na wiek*		
Abnormalities registered in the 24-hour Holter ECG *Nieprawidłowości rejestrowane w 24-godzinnym zapisie EKG metodą Holtera*	Patients over the age of 5 (36 children) *Pacjenci powyżej 5 r.ż. (36 dzieci)*	Patients under the age of 1 (9 children) *Pacjenci poniżej 1 r.ż. (9 dzie*ci)
Premature Ventricular Contractions *Przedwczesne pobudzenia komorowe*	33.3%	11.1%
Premature Atrial Contractions *Przedwczesne pobudzenia komorowe*	33.3%	−
Sinus tachycardia *Tachykardia* *zatokowa*	13.3%	−
Sinoatrial II*°* block *Blok zatokowo-przedsionkowy II°*	6.7%	−
Atrioventricular II*°* block type 2:1 Blok przedsionkowo-komorowy II⍰ typu 2:1	2.2%	−
Atrioventricular reentrant tachycardia *Nawrotny częstoskurcz przedsionkowo-komorowy*	2.2%	−

Based on ECG and on 24-hour Holter ECG, 10 children (22.2%) were diagnosed with the intermittent type of Wolff-Parkinson-White syndrome. 1 child (2.2%) had a latent type of Wolff-Parkinson-White syndrome and the rest of the study group (75.6%) had typical features of preexcitation present during sinus rhythm (apparent type of preexcitation) (Table I).

Based on echocardiography, 4 children (8.9%) were diagnosed with structural heart defects, which included atrial septal defect (ASD) in 2 children, ventricular septal defect (VSD) in 1 child and corrected transposition of great arteries (L-TGA) in 1 child.

For the vast majority of children over the age of five years, the cycloergometric exercise test using the James protocol was negative. It had a positive outcome in 4 children (8.9%). In these cases, physical exercise caused atrioventricular reentrant tachycardia or generated premature ventricular contractions. In 2 children (4.4%) the loss of preexcitation was noticed at the peak of exercise.

**Fig. 3:**
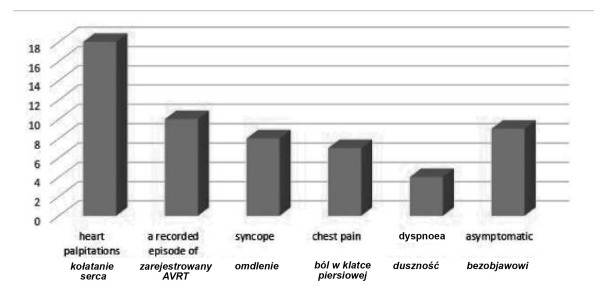
Symptoms presented by children in the group over the age of five. Ryc. 3. Objawy prezentowane przez dzieci w grupie powyżej piątego roku życia.

The symptoms presented by the children varied depending on age. 29 patients (64.4%) were admitted to the Department of Pediatric Cardiology as planned admissions, and 16 children (35.6%) were admitted to hospital as emergencies. The most common symptom in the group of children over five years of age were heart palpitations, which occurred in 18 patients (50%). 8 children (22.2%) reported a history of syncope or loss of consciousness. 7 children (19.4%) reported to the Emergency Room because of chest pain. 4 children (11.1%) experienced dyspnoea. Moreover, 8 children (22.2%) reported previously mentioned symptoms during physical exercise or stress. 9 children (25.0%) were asymptomatic and were diagnosed based on an incidental ECG recording. In 10 children (27.8%) the first symptom of the Wolff-Parkinson-White syndrome was atrioventricular reentrant tachycardia registered on a 12-lead ECG (Figure 3).

In 88.9% of the group of children under the age of one year, the first manifestation of Wolff-Parkinson-White syndrome was atrioventricular reentrant tachycardia. In 3 infants it was accompanied by nonspecific symptoms, such as apathy or loss of appetite. In 33% AVRT occurred in the course of infection.

Prior to diagnosis, 11 patients (30.6%) in the group of children older than five years actively participated in sports. 2 of them were diagnosed by a sports medicine doctor. 2 other children were qualified for active participation in sports despite the presence of typical features of preexcitation in ECG. 7 patients were not supervised by a sports medicine doctor.

In the group of older children the mean duration of symptoms before the diagnosis was 2.5 years.

Due to differences in treatment, the study group was divided according to age into children under one year of age and children over five years of age. 19 children (42.2%) required the implementation of antiarrhythmic therapy in the form of β-blocker agents. 8 infants (88.9%) received propranolol and 4 (44.4%) of them required additional amiodarone therapy because of the insufficient effect of propranolol. 1 infant did not require antiarrhythmic treatment due to the lack of atrioventricular reentrant tachycardia.

11 children (30.6%) in the group of patients older than five years received metoprolol, whereas in 2 cases (5.6%) propafenone was additionally incorporated into the treatment.

**Fig. 4:**
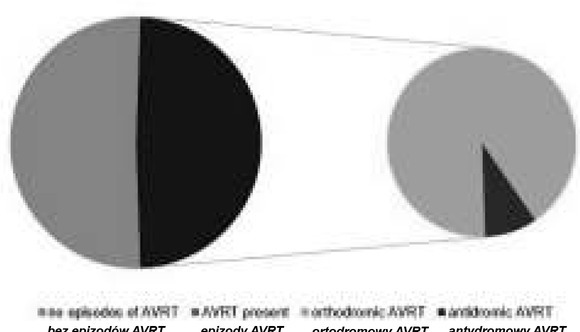
Occurrence of atrioventricular reentrant tachycardia (AVRT) in the study group. Ryc. 4. Występowanie częstoskurczu nawrotnego przedsionkowo--komorowego w badanej grupie.

**Fig. 5:**
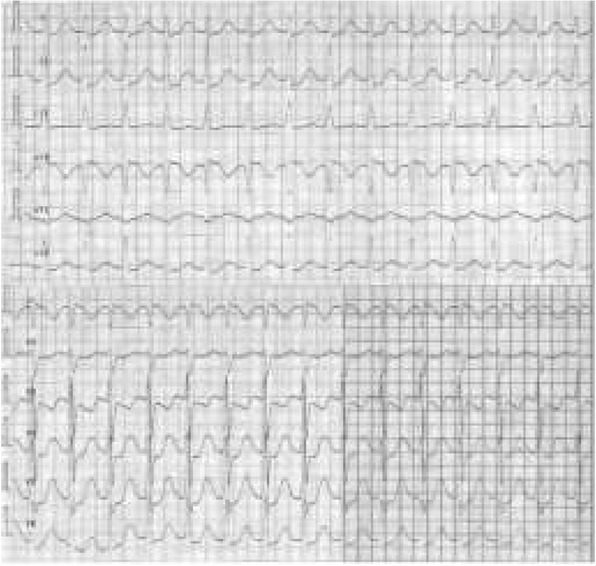
ECG of a 9-year-old patient with orthodromic atrioventricular recurrent tachycardia. Heart Rate = 240 bpm [own material]. Ryc. 5. EKG 9-letniego pacjenta z ortodromowym nawrotnym częstoskurczem przedsionkowo-komorowym. Rytm serca o częstości 240/min [materiał własny].

22 children (48.9%), including 14 patients from the group over the age of five years and 8 from the group younger than one year, had at least one episode of atrioventricular reentrant tachycardia. 20 patients (90.9%) had orthodromic atrioventricular reentrant tachycardia and 2 patients (9.1%) had antidromic atrioventricular reentrant tachycardia (Figure 4, 5).

The most effective termination method of atrioventricular reentrant tachycardia in the group of children under the age of one year was the administration of a rapid bolus of adenosine (i.v.), which was effective in 5 children (62.5%). In 2 children (25.0%) atrioventricular reentrant tachycardia was terminated by amiodarone administered according to the standards.

**Fig. 6:**
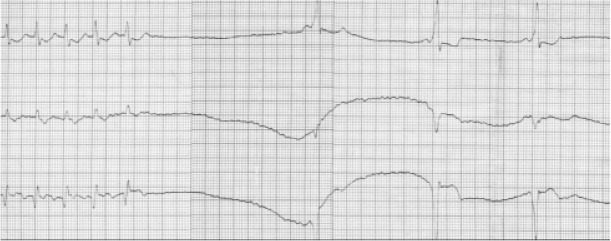
Termination of atrioventricular reentrant tachycardia in response to administration of adenosine. Heart rate = 300 bpm [own material]. Ryc. 6. Przerwanie nawrotnego częstoskurczu przedsionkowo-komorowego w odpowiedzi na podaż adenozyny. Rytm serca o częstości 300/min [materiał własny].

In the group of children over five years of age, 5 children (35.7%) had spontaneous termination of atrioventricular reentrant tachycardia, in 3 children (21.4%) Valsalva maneuvers proved sufficient, and 6 patients (42.9%) required the administration of adenosine (Figure 6).

**Fig. 7:**
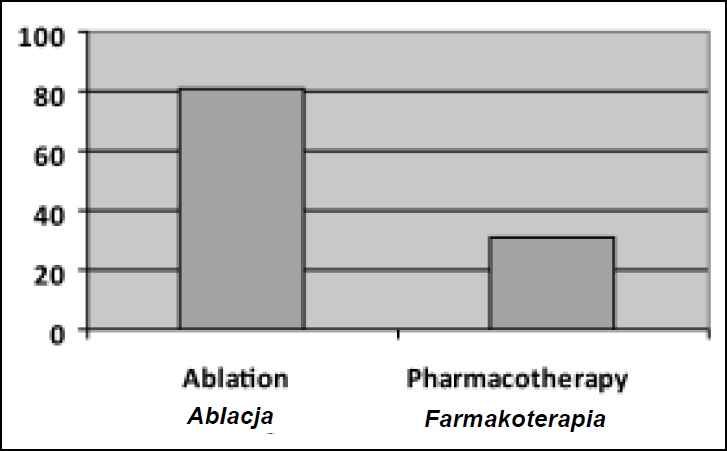
Forms of treatment applied in children over the age of five. Ryc. 7. Zastosowane metody leczenia u dzieci powyżej piątego roku życia.

Ablation was a recommended form of treatment for 29 children in the group of patients over the age of five years (80.6%). 20 children (68.9%) underwent ablation, 16 children (80.0%) underwent radiofrequency ablation (RF) and 4 patients (20.0%) underwent cryoablation. 4 children (13.8%) still await ablation. 5 children (17.2%) refrained from this form of therapy due to the lack of parental consent (Figure 7).

**Fig. 8:**
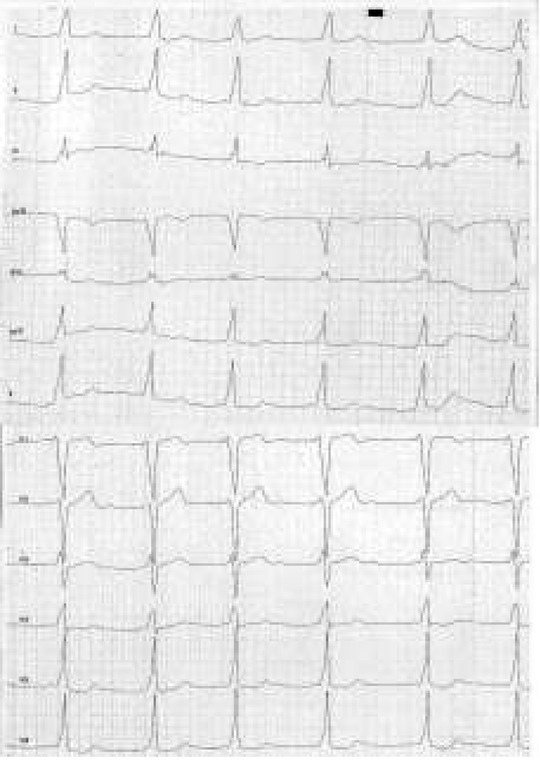
ECG of a 9-year-old patient with Wolff-Parkinson-White Syndrome. Sinus rhythm, heart rate = 85 bpm, normal cardiac axis, P-R interval =80 ms, QRS complex= 140 ms, J-T interval =300 ms, delta wave on an ascending arm of a QRS complex. Electrophysiological study prior to ablation confirmed presence of bilateral right-sided and anteroseptal accessory pathway [own material]. Ryc. 8. EKG 9-letniego pacjenta z zespołem Wolffa-Parkinsona-White’a. Rytm zatokowy, o częstości 85/min, pośrednia oś serca, odstęp P-R = 80 ms, QRS = 140 ms, odstęp J-T = 300 ms, fala delta na ramieniu wstępującym zespołu QRS. Badanie elektrofizjologiczne przed ablacją potwierdziło obecność drogi dodatkowej prawostronnej oraz przednio-przegrodowej [materiał własny].

Electrophysiological mapping analysis prior to ablation showed that within the group of patients older than five years, 12 children (60.0%) had right-sided accessory pathways, 5 children (25.0%) had left-sided accessory pathways, 2 children (10.0%) had anteroseptal accessory pathways and 1 child (5.0 %) had a midseptal accessory pathway (Figure 8).

According to the standards, after the procedure of ablation, all the children received 75 mg of aspirin for one month. All the children underwent a two-year follow-up after the ablation.

Ablation proved to be a completely effective form of therapy in 90% of the children. In 10% (2 children) of the patients after the ablation, residual conduction through the accessory pathway was visible in the form of discrete features of preexcitation periodically recorded in ECG. However, the recurrence of atrioventricular reentrant tachycardia episodes was not observed.

## Discussion

Preexcitation syndrome is a congenital anomaly associated with the presence of an abnormal accessory pathway conducting electrical impulses between the atria and the ventricles, bypassing the atrioventricular node. It affects 0.1-0.3% of the global population, more often males [1]. The number of diagnosed asymptomatic patients with preexcitation syndromes is gradually increasing, which is associated with a more frequent use of ECG as a diagnostic tool in the pediatric population [2, 3]. 45 children diagnosed with preexcitation syndrome, 62% of whom were males, were hospitalized in the Department of Pediatric Cardiology over a 7-year period. Most of the study group were diagnosed with Wolff-Parkinson-White syndrome, which is the most common type of preexcitation syndrome in children [4].

The study group was further divided into a group of children younger than one year and a group of patients over five years of age, considering the symptoms, clinical course, diagnostic procedures and treatment. About 60% of the children with atrioventricular reentrant tachycardia present symptoms of arrhythmia in the first year of life, mostly during the first 3-4 months. In most patients the risk of supraventricular tachycardia decreases with age as a result of changes in the electric properties of the accessory pathways (loss of preexcitation) [1]. It is estimated that 20-37% of infants with preexcitation syndrome have concomitant heart defects [2]. The most common are: Ebstein’s anomaly, mitral valve prolapse, atrial septal defect, transposition of great arteries, coarctation of the aorta, dextrocardia and ventricular septal defect [5]. In the study group 3 infants (33% of the children under 1 year of age) had congenital heart defects in the form of atrial septal defect, ventricular septal defect or congenital corrected transposition of great arteries. 1 patient had a family history of preexcitation syndrome.

Recent studies led to the discovery of the autosomal dominant form of Wolff-Parkinson-White syndrome associated with a gene located on the long arm of chromosome 7. Research shows that 3% of the patients with Wolff-Parkinson-White syndrome have symptomatic first-degree relatives [1].

Non-invasive diagnostic tools (ECG, 24-hour Holter ECG, exercise test) can determine whether the typical features of preexcitation disappear during physical exercise or not. The loss of preexcitation can indicate that the accessory pathway does not conduct impulses faster than the physiological conductive system, which can indicate a lower risk of episodes of atrioventricular reentrant tachycardia [6]. Exercise may enhance accessory pathway conduction and adds further value in the noninvasive assessment of the WPW pattern. Only abrupt and complete loss of preexcitation during exercise testing confirms a long anterograde pathway refractory period and, hence, a low risk profile. The persistence of preexcitation during exercise stress predicts the shortest preexcited R-R interval in atrial fibrillation <250 ms or an anterograde refractory period of accessory pathway <250 ms in electrophysiological study [7]. Recent guidelines recommend invasive evaluation for asymptomatic patients when the pre-excitation does not disappear during exercise testing. Exercise testing has a high negative predictive value only [8]. In the study group abrupt disappearance of preexcitation at the peak of the exercise test occurred in 2 children (4.4%). However, in one patient, although there was an abrupt loss of preexcitation, an episode of orthodromic atrioventricular reentrant tachycardia occurred subsequently.

The presence of typical preexcitation features on a 12-lead ECG recording can be found in 1.5-3.1 per 1,000 people. The actual incidence of preexcitation syndromes may be higher, because not every patient receives ECG. Moreover, features of preexcitation are not visible on ECG in some of the cases. This may be associated with a latent or intermittent type of Wolff-Parkinson-White syndrome. In such patients a proper diagnosis of Wolff-Parkinson-White syndrome can be set only after the occurrence of an episode of atrioventricular reentrant tachycardia [1]. In the study group almost half of the patients had a documented episode of atrioventricular reentrant tachycardia.

29% of the children in the study group had left axis deviation. In a 1990 article it was recognized as one of the three main features visible in an ECG recording of approximately 33% of patients with preexcitation syndromes [9].

In older patients, accessory pathways are usually apparent, which means that the typical features of preexcitation can be found in a standard (resting) ECG recording [10]. In the study group, 75.6% of children had apparent preexcitation characteristics, which enabled the diagnosis of preexcitation syndrome based on ECG.

Intermittent preexcitation syndrome is one in which the features of preexcitation are not always visible in ECG but appear under certain circumstances (e.g. during physical exercise) [6]. This type of preexcitation was present in 22% of children in the study group. Patients with intermittent preexcitation syndrome typically exhibit slower descending conduction through the accessory pathway, and thus have a lower risk of sudden cardiac death [6]. A recent expert consensus document on the management of asymptomatic patients suggested that invasive measurement of the shortest pre-excited R–R interval (SPERRI) in AF is useful for risk stratification, and patients with a SPERRI ≤250 ms are at increased risk of sudden cardiac death [11].

Other arrhythmias, such as premature atrial or ventricular beats, are quite common in patients with preexcitation syndromes and may lead to episodes of atrioventricular reentrant tachycardia [12]. In the group of patients presented, such heart rhythm disorders were recorded in more than 60% of the children.

Only 50% of the patients with preexcitation syndromes present clinical symptoms. The most common manifestation are heart palpitations, mostly associated with the occurrence of atrioventricular reentrant tachycardia [1]. In a study on a group of 212 patients with Wolff-Parkinson-White syndrome who experienced heart palpitations, 64% had episodes of atrioventricular reentrant tachycardia [4]. Similar results were obtained in the presented study group, in which the most common reason to seek medical advice were symptoms accompanying supraventricular tachycardia, such as heart palpitations or syncope. On the other hand, during tachyarrhythmia, infants can present nonspecific symptoms, such as irritability, loss of appetite, gastrointestinal or respiratory problems, anxiety or even cardiogenic shock [1]. 33% of the infants in the study group presented unusual symptoms, such as apathy or loss of appetite.

In infants the diagnosis is usually based on a documented episode of atrioventricular reentrant tachycardia. In the study group, as many as 90% of the infants had an episode of atrioventricular reentrant tachycardia. Although the frequency of episodes of tachycardia is reduced after the first year of life (in 90% of the patients), 30% of the children have relapsing episodes at the age of 7-8 years. It is believed that in more than 40% of the infants, accessory pathways lose their ability to conduct impulses [6]. In the study group 1 child, who experienced atrioventricular reentrant tachycardia as an infant, had recurrent episodes of supraventricular tachycardia at an early school age.

A publication by Cohen et al. shows that if the features of preexcitation in ECG coexist with heart palpitations in children over the age of 5 years, 75% of those children will continue to suffer from them over the next 10 years [6].

After 57 months of research conducted on a group of asymptomatic pediatric patients aged 8-12 years who had ECG and 24-hour Holter ECG recordings monitored every 6 months, 51 out of 133 children developed clinical symptoms of preexcitation, and 19 of them had an incident of a potentially life-threatening arrhythmia [1]. 25% of the children in the study group did not present any symptoms of preexcitation prior to diagnosis.

In the population of patients with Wolff-Parkinson-White syndrome, the most common type of tachycardia is orthodromic atrioventricular tachycardia [12, 13]. It was also a dominant type of tachycardia in the study group; only 2 children (9%) had a documented episode of antidromic reentrant tachycardia. In 10% of the patients with preexcitation, the presence of an accessory pathway is a risk factor of a sudden cardiac death [12]. The risk is particularly high for patients with accessory pathways which have a short refraction period [14]. It is therefore important to diagnose and treat this group of patients as early as possible.

The guidelines of the European and American Heart Associations concerning young asymptomatic athletes with preexcitation syndromes require that such patients should undergo electrophysiological testing to evaluate the risk of a sudden cardiac death and that physical exercise should be discontinued until the end of the diagnostic process [6]. As many as 30% of the children in the study group participated in sports whilst being diagnosed. Some of them trained despite the features of preexcitation visible in ECG but overlooked or ignored by their sports medicine doctor.

In our study group, the mean duration of symptoms prior to diagnosis was 2.5 years. Such a long period of time might be the result of a lack of specific symptoms or the negligence of symptoms by some patients. Besides, not all of the children were referred to ECG examination despite clinical indications.

Electrophysiological studies conducted in the population of adult patients with preexcitation syndromes show a prevalence of left-sided accessory pathways (50%); septal accessory pathways are less frequent (30%) and right-sided accessory pathways are diagnosed in 20% of patients with WPW syndrome [2,5]. In the group of older children who underwent electrophysiological testing, 60% had right-sided accessory pathways, 25% had left-sided accessory pathways and 15% had septal accessory pathways. In the case of right-sided pathways the time in which an impulse reaches the accessory pathway is shorter, since the distance from the sinus node to the accessory pathway is shorter than to the sinoatrial node, which is reflected in ECG as more distinct features of preexcitation [15]. The location of an accessory pathway is important because of a subsequent risk of complications and recurrences after ablation. This particularly applies to accessory pathways located in the front part of the septum and in patients with structural heart defects and with more than one accessory pathway [6].

In the study group, the most effective method of terminating an episode of supraventricular tachycardia was the administration of adenosine. Studies conducted in the year 2000 on a group of patients under the age of 18 with reentrant supraventricular tachycardia, showed that adenosine restored sinus rhythm in 79% of the cases [16].

In 1/5 of the study group sinus rhythm was restored by the application of vagal maneuvers in the form of Valsalva maneuver. This coincides with the results of research done in 2013 where, among 316 patients with supraventricular tachycardia, Valsalva maneuver was efficient in 19% of the group [17].

β-blockers are commonly used to treat patients with preexcitation syndromes and with atrioventricular reentrant tachycardia. Their effectiveness is rated from 50% to 90%. In the pediatric population the most commonly used β-blockers are propranolol and metoprolol [16]. In the group presented, 89% of the infants received propranolol, while 30.6% of older children required the administration of metoprolol. Nearly half of the infants in the study group required an addition of amiodarone to the β-blocker therapy.

In their research of 2001 Etheridge et al. showed that in their study group of newborns and infants the application of amiodarone or of the combination of propranolol and amiodarone was effective in all of their patients [16]. The literature shows that propafenone also has a role in the treatment of tachycardia as a second-line therapy, when a first-line treatment does not bring the expected results [16]. A combination of propafenone and β-blocker was applied in 2 children in the group of children over 5 years of age.

The guidelines state that in children over the age of 5 years with WPW syndrome and with recurrent and/ or symptomatic SVT, catheter ablation is a class I, level C recommendation. The recommended antiarrhythmic therapy in this group of children includes flecainide, propafenone and sotalol as class I recommendation, as well as amiodarone as class II b recommendation. For children under the age of 5 years the guidelines indicate flecainide and propafenone as class I level C recommendation, sotalol as class IIa recommendation and catheter ablation as well as amiodarone as a class IIb recommendations. For asymptomatic children under and over the age of 5 years the guidelines indicate ablation as class III and IIb, respectively, and an antiarrhythmic therapy as class III recommendation for both groups [18].

Ablation of the accessory pathway is considered the most effective method of treatment of preexcitation syndromes. It is a preferred method for symptomatic patients with a high risk of sudden cardiac death who meet the age requirement [14]. Due to the formation of postoperative scars which are relatively large in relation to the size of the heart, and due to a high risk of a third-degree atrioventricular block, ablation in children under the age of two years is considered only as a life-saving treatment. Most medical centers offer ablation as a treatment of choice for children older than 6-10 years. In older children, the prophylactic antiarrhythmic drug therapy is recommended in the case of a patient’s preference - while waiting for ablation, after failed ablation, and whenever catheter ablation is judged to be associated with too high a risk of complications [18]. However, some authors believe that this treatment can be used alongside pharmacological treatment in children weighing more than 15 kgs [5]. Age <4 years or weight <15 kg are independent risk factors for complications associated with the procedure. Therefore, in children younger than 5 years, drug treatment is recommended as an initial management of recurrent AVRT, due to the age-related increased risk of catheter ablation and considering the natural course with spontaneous remissions of AVRT [18].

In patients older than 5 years, with episodes of supraventricular tachycardia associated with preexcitation syndrome, the recurrence rate of atrioventricular reentrant tachycardia is 75%, which is why most of the medical centers offer ablation as a primary form of therapy in this group of patients [6]. In the study group, 81% of patients over the age of 5 years were qualified for the procedure. 69% of those children underwent ablation. 80% underwent radiofrequency ablation (RF) and 20% underwent cryoablation. Cryoablation is widely used in pediatric practice due to the increased safety of the treatment, especially in patients with accessory pathways located near the septum or near the coronary sinus [6]. In the study group, it was a method of choice in 20% of the patients who underwent ablation. They were mainly patients with an anteroseptal accessory pathway.

The recurrence of atrioventricular reentrant tachycardia after a successfully performed ablation of the accessory pathway in young patients is estimated at about 11% and usually occurs within the first 2 months after the procedure. The Pediatric Electrophysiology Society conducted a study in which the efficacy of ablation in children with Wolff-Parkinson-White syndrome equaled 93% [4, 6]. In the study group, the treatment was effective for 90% of children, while in 10% of the patients residual conduction through the accessory pathway was visible in ECG; these patients, however, have not experienced any episodes of atrioventricular reentrant tachycardia post-ablation.

## Conclusions

1. Heart palpitations are the most common symptom of preexcitation syndrome in the group of children and adolescents studied.
2. Orthodromic atrioventricular reentrant tachycardia is the most frequently recorded arrhythmia in WPW syndrome.
3. 25% of pediatric patients with preexcitation are diagnosed based on an incidental ECG recording.
4. In the group of children over five years of age ablation of the accessory pathway is a recommended form of treatment.
5. In children under the age of one year preventive pharmacological treatment is applied for 6 to 12 months.
